# Supplementary material for: The anti-sigma factor RsrA responds to oxidative stress by reburying its hydrophobic core
Source: Nat Commun. 2016 Jul 19;7:12194. doi: 10.1038/ncomms12194 (PMC4960319; doi:10.1038/ncomms12194)
Supplement: Supplementary Information — Supplementary Figures 1-8 and Supplementary Tables 1-2. [file ncomms12194-s1.pdf]

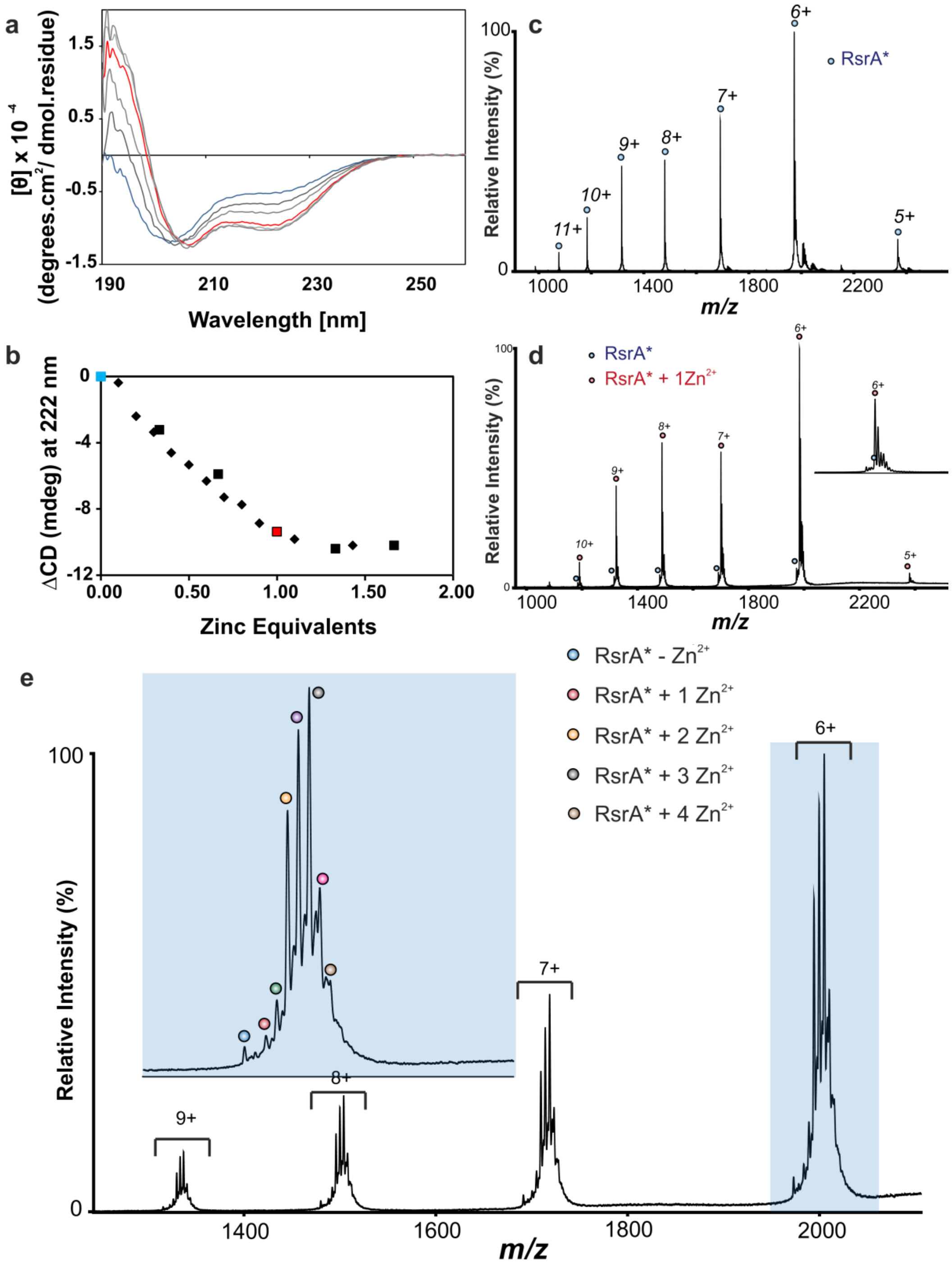


**Supplementary Figure 1. Zinc binding to RsrA.** ***a***, Far-UV CD spectra of RsrA* incubated with increasing concentrations of zinc (in 10 mM Tris pH 7.5, at 20˚C) starting from apo-protein (*blue spectrum*). Wild type RsrA yielded poor CD spectra and so zinc titrations were performed with RsrA*. The protein becomes more helical as the amount of zinc increases but saturates at one equivalent of zinc (*red spectrum*). ***b***, Figure shows variation of RsrA* mdeg at 222 nm starting from apo-RsrA* (*blue square*, equivalent to blue spectrum in *a*) with increasing zinc concentration. *Red square* is equivalent to the red spectrum in *a*. ***c***, Native-state ESI-MS spectrum of apo-RsrA* (same sample used in CD experiment, blue point in *a*). ***d***, Native-state ESI-MS spectrum of RsrA* incubated with 1 equivalent of zinc (same sample used in CD experiment, red point in *a*). Protein was buffer exchanged into 20 mM ammonium acetate before data collection (theoretical/observed masses for apo- and single zinc bound to RsrA* are 11834/11832.82 ± 0.57 and 11897/11896.75 ± 1.02, respectively). ***e***, Stoichiometry of zinc binding to wild type RsrA. Figure shows the high resolution native-state ESI-MS spectrum of RsrA incubated with excess zinc. 10 μM RsrA was dissolved in 50 mM Tris HCl pH 7.5, 100 mM NaCl, 0.1 mM $ZnCl_2$ and 10 mM DTT. Samples were buffer exchanged into 20 mM ammonium acetate before measurements were made. Spectrum shows up to 3 $Zn^{2+}$ ions bind to RsrA. Theoretical and observed masses for apo, $1Zn^{2+}$, $2Zn^{2+}$ and $3Zn^{2+}$ bound samples were 11962.3/11957.08 ± 4.90 Da, 12025.3/12022.72 ± 6.21 Da, 12088.3/12087.36 ± 5.36 Da and 12151.3/12154.20 ± 4.46 Da respectively. Similar data were obtained for RsrA*, where all non-essential cysteines (Cys3, Cys31, Cys61 and Cys62) were mutated to alanine, indicating that non-stoichiometric zinc binding is not associated with cysteine residues outside of the ZAS sequence motif.

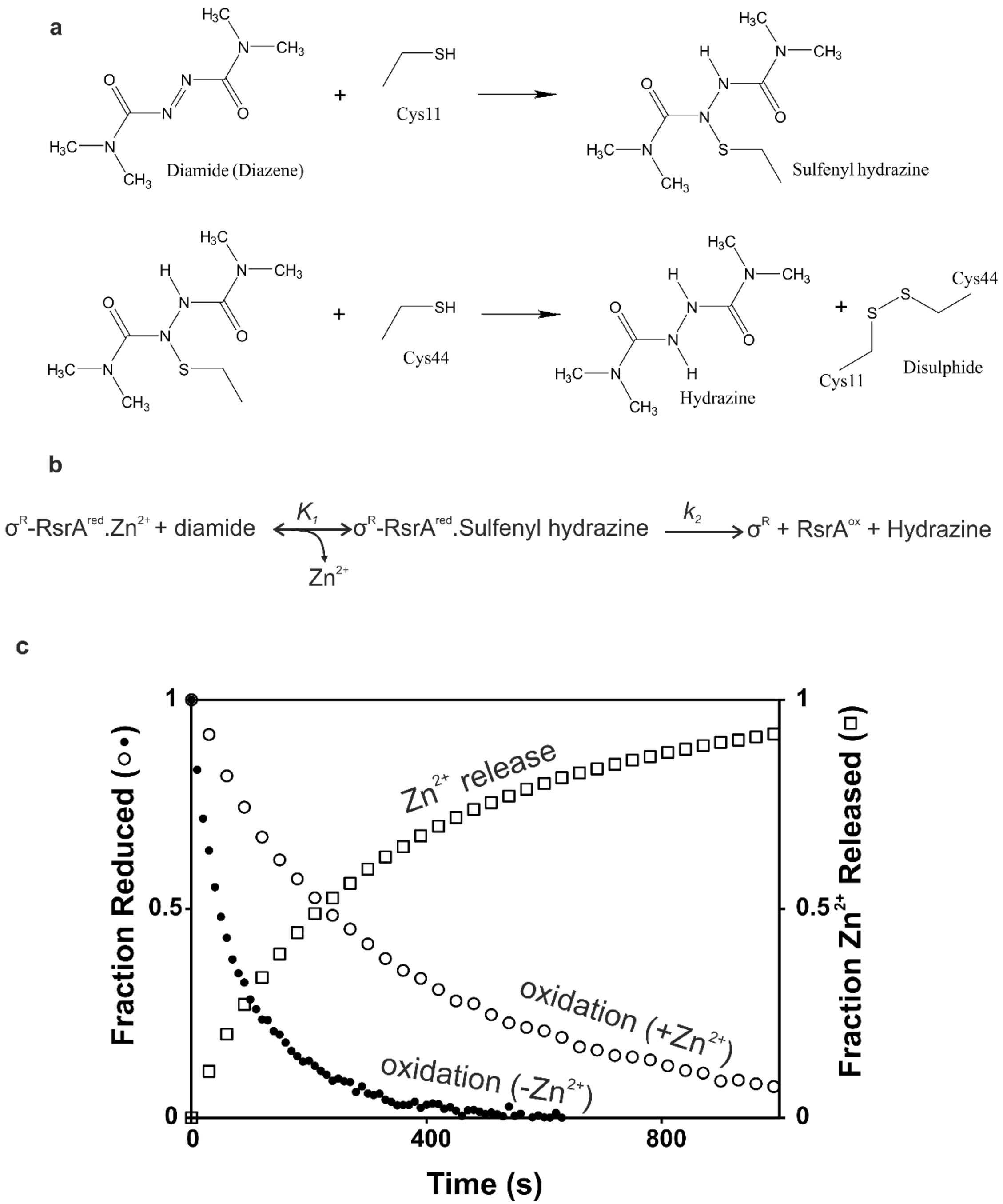


**Supplementary Figure 2. Diamide oxidation kinetics.** ***a*****,** Schematic showing the chemical conversion of diamide from the diazene to hydrazine forms during the oxidation of RsrA. Also shown is the sulfenyl hydrazine intermediate. Spectroscopic measurement of diamide reduction by RsrA exploited the absorption of diamide at 320nm. The product hydrazine does not absorb at this wavelength. ***b*****,** Kinetic mechanism for diamide-induced oxidation of RsrA$^{red}$.Zn$^{2+}$. The mechanism is based on data obtained from diamide reduction kinetics (main text, **Figure 4b**). Values for $K_1$ and $k_2$ obtained from the data shown in Figure 4b were 0.7 mM and 0.15 $s^{-1}$, respectively. ***c*****,** Kinetics of RsrA$^{red}$.Zn$^{2+}$ oxidation by diamide are the same as for the RsrA$^{red}$.Zn$^{2+}$-σ$^R$ complex. Experiments were conducted at 25˚C in 50 mM Tris HCl pH 7.5 buffer containing 100 mM NaCl. Proteins were first reduced with DTT (10 mM) then buffer exchanged to remove the reducing agent. Where indicated, stoichiometric zinc was added. Oxidation of and zinc release from RsrA$^{red}$.Zn$^{2+}$ on treatment with diamide under second-order conditions (25 μM) was measured spectrophotometrically (see Materials and Methods for further details). The fraction of reduced RsrA$^{red}$.Zn$^{2+}$ was determined by monitoring the change in diamide absorbance at 320 nm in the presence and absence of stoichiometric zinc (*open and closed circles*, respectively). Zinc release (*open squares*) was monitored at 500 nm using the PAR assay. For clarity, only every 80$^{th}$ data point is shown in these curves. The two methods showed good agreement for the bimolecular rate constant for diamide-induced oxidation of RsrA$^{red}$.Zn$^{2+}$ (196 ± 12.3 $M^{-1}$ $s^{-1}$and 194 ± 29 $M^{-1}$ $s^{-1}$, respectively). Oxidation of RsrA was five-fold faster in the absence of zinc (946 ± 24 $M^{-1}$ $s^{-1}$). These data closely mirror those obtained for RsrA bound to σ$^R$ indicating that complex formation does not influence redox sensing by RsrA.

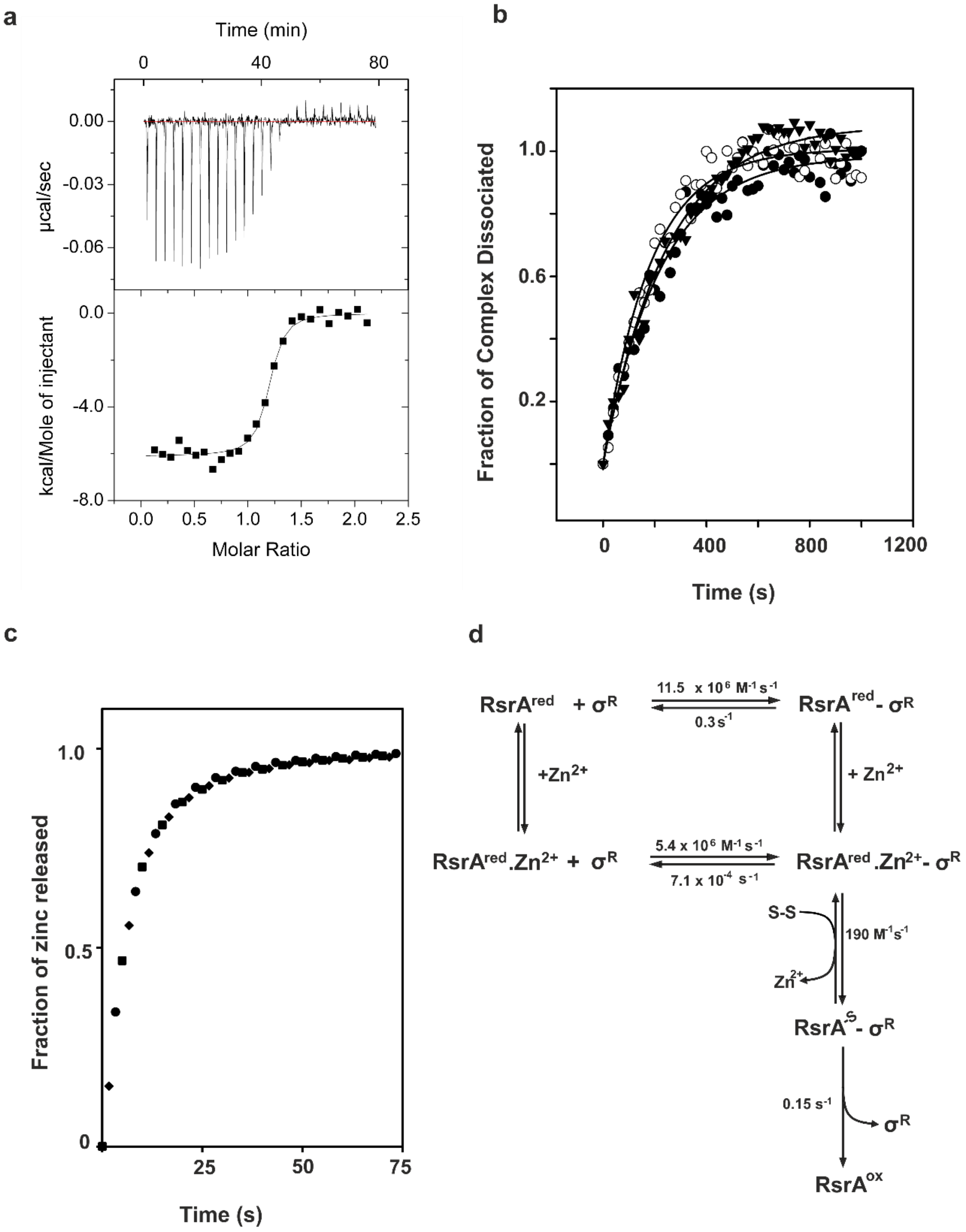


**Supplementary Figure 3. Additional $Zn^{2+}$ ions do not affect the rate of RsrA oxidation.** ***a***, Competition ITC data for $\sigma^R$ Trp88Ile Trp119Ile binding $RsrA^{red}.Zn^{2+}$, as described for the wild type complex (main text, **Figure 3a**). Fitted parameters for a single binding site model were N = 1.17 ± 0.09, $K_d$ = 0.173 ± 0.034 nM, ΔH = -20.34 ± 0.91 kcal/mol. ***b***, Tryptophan emission fluorescence spectroscopy was used to determine the kinetics of $RsrA^{red}$-$\sigma^R$ oxidative dissociation on treatment with 10 mM $H_2O_2$ and increasing zinc stoichiometry. Oxidation induced dissociation of the $RsrA^{red}$-$\sigma^R$ complex was the same regardless of whether 1 (*open circles*), 2 (*closed triangles*) or 3 (*closed circles*) equivalents of zinc, respectively, were added to the protein. ***c***, The rate of $Zn^{2+}$ release from the $RsrA^{red}.Zn^{2+}$-$\sigma^R$ complex on treatment with 15 mM diamide is the same when RsrA is bound with 1 (*diamonds*), 2 (*circles*) or 3 (*squares*) equivalents of zinc, respectively. Hence, increasing zinc stoichiometry does not influence redox sensing by the $RsrA^{red}.Zn^{2+}$-$\sigma^R$ complex. *d*, Kinetic scheme of RsrA-$\sigma^R$ interaction. ***d***, Kinetic scheme showing RsrA-$\sigma^R$ protein-protein interactions and the influence of zinc and oxidant. Rate constants are taken from Figures 4 and 5. The kinetics of $RsrA^{red}.Zn^{2+}$-$\sigma^R$ oxidation are those for a disulfide-containing oxidant, denoted by S-S in the scheme and mimicked by diamide in our experiments, to form a mixed disulfide ($RsrA^{-S}$), which we infer (Figure 4b). The oxidation process is a two-step reaction involving the slow loss of zinc followed by a fast step involving dissociation of $\sigma^R$ and formation of the trigger disulfide bond (Cys11-Cys44 or Cys11-Cys41).

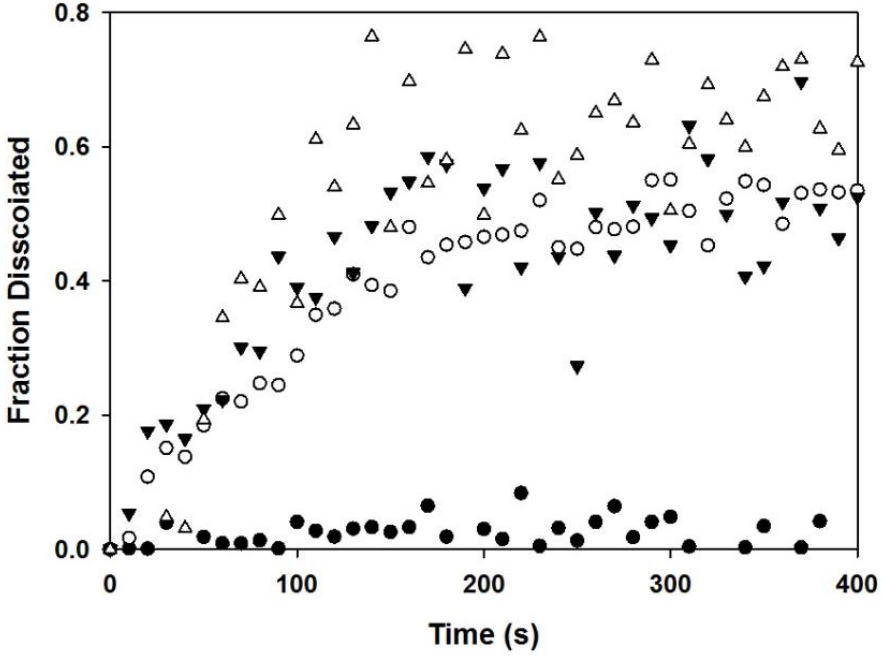


**Supplementary Figure 4. $H_2O_2$-induced dissociation of RsrA histidine mutants confirms the redundancy of the trigger disulfide bond**. Comparison of $H_2O_2$ (10 mM) induced dissociation of RsrA*$^{red}$.$Zn^{2+}$-$\sigma^R$ complex (*open circles*) with RsrA*$^{red}$.$Zn^{2+}$ Cys11His-$\sigma^R$ (*closed circles*), RsrA*$^{red}$.$Zn^{2+}$ Cys41His-$\sigma^R$ (*closed triangles*) and RsrA*$^{red}$.$Zn^{2+}$ Cys44His-$\sigma^R$ (*open triangles*) complexes. The data emphasise the redundancy of the trigger disulfide bond in RsrA in which either Cys41 or Cys44 can form a disulfide with Cys11 to release $\sigma^R$.

**a**

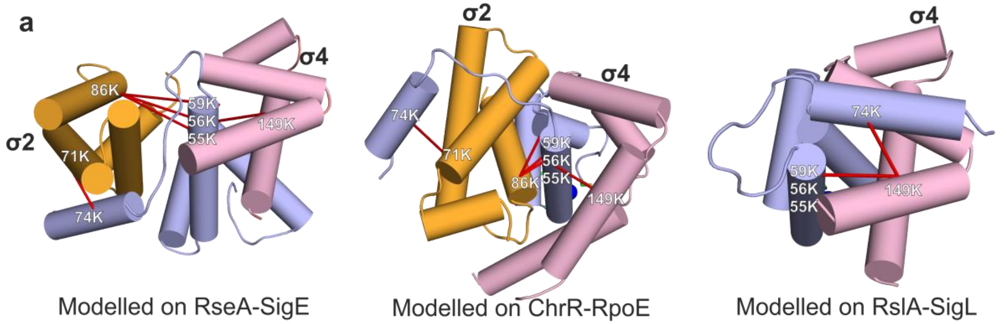


| Model | X-walk distance (Solvent Accessible Surface Distance Å) | | | | | |
|---|---|---|---|---|---|---|
| | RseA-$\sigma^E$ | | ChrR-RpoE | | RslA-$\sigma^L$ | |
| Observed Cross links (BS3=✓, BS2G=✓) | Distance | Cross link expected | Distance | Cross link expected | Distance | Cross link expected |
| RsrA56K-σR86K ✓✓ | 15.6 | ✓ | 4.4 | ✓✓ | NK | |
| RsrA55K-σR86K ✓✓ | 22.4 | × | 9.6 | ✓✓ | NK | |
| RsrA59K-σR86K ✓✓ | 19.3 | × | 5.7 | ✓✓ | NK | |
| RsrA74K-σR71K ✓✓ | 22.7 | × | 14.1 | ✓✓ | NK | |
| RsrA74K-σR86K ✓✓ | >50 | × | >50 | × | NK | |
| RsrA74K-σR149K ✓✓ | >50 | × | >50 | × | 6.2 | ✓✓ |
| RsrA56K-σR149K ✓ | 15.8 | ✓ | 8.5 | ✓✓ | 15.8 | ✓ |

**b**

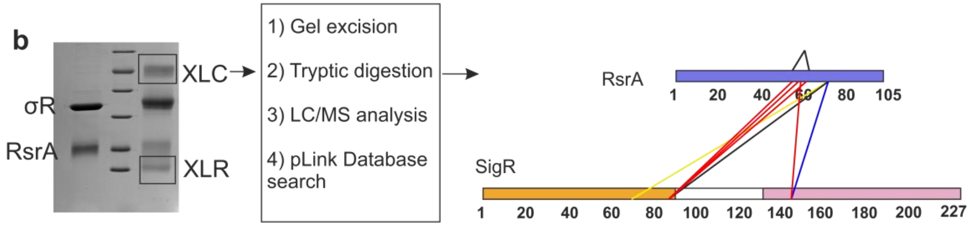


**c**

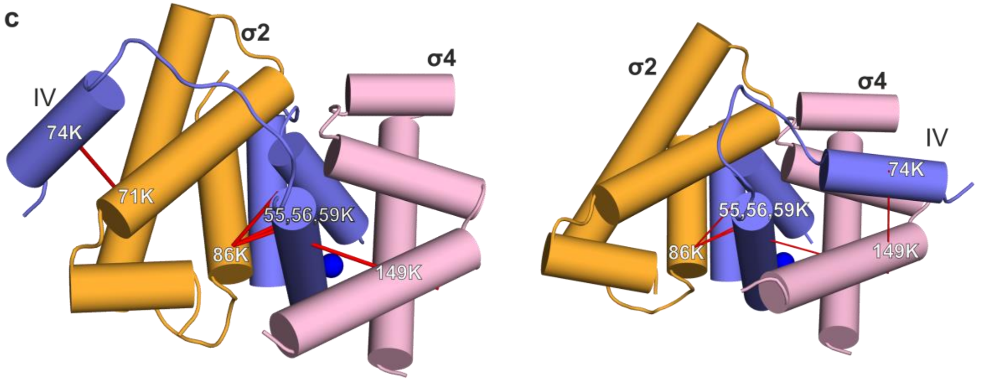


**Supplementary Figure 5. Cross-linking based homology model of the RsrA$^{red}$.Zn$^{2+}$-σ$^{R}$ complex.** ***a*,** The RsrA$^{red}$.Zn$^{2+}$-σ$^{R}$ complex (RsrA, blue; σ$^{R}$ σ$^{2}$ domain, orange, and σ$^{4}$ domain, pink) was modelled on three anti-sigma factor-sigma factor complexes, *E. coli* RseA-σ$^{E}$ (sequence identity 15.8%, pdb code 1OR7), *R. sphaeroides* ChrR-RpoE/σ$^{E}$ (sequence identity 11.1%, pdb code 2Q1Z) and *M. tuberculosis* RslA-σ$^{L}$ (sequence identity 20.6%, pdb code 3HUG). Only ChrR and RslA are ZAS proteins but all three have the so-called ASD fold. The structure of the RslA-σ$^{L}$ complex only contains half of the sigma factor (σ$^{4}$ domain) whereas the other complexes have both σ$^{2}$ and σ$^{4}$ domains. Red lines indicate the lysine-specific crosslinks observed for the RsrA$^{red}$.Zn$^{2+}$-σ$^{R}$ complex mapped onto each model. The table beneath the structures shows the solvent accessible surface distances for lysines in the RsrA$^{red}$.Zn$^{2+}$-σ$^{R}$ complex predicted for the different models and summarizes the cross-links observed for the different homo-bifunctional cross-linkers used (BS3, bis(sulfosuccinimidyl)suberate, 11.4 Å cross-linking distance; BS2G, bis(sulfosuccinimidyl) 2,2,4,4-glutarate), 7.7 Å cross-linking distance. NK, not known. The cross-links observed in the RsrA$^{red}$.Zn$^{2+}$-σ$^{R}$ complex (*left hand panel*) indicated the complex was most similar to the ChrR-RpoE complex but also had similarities to the RslA-σ$^{L}$ complex. ***b***, Strategy used to obtain lysine-specific cross-linking data for the RsrA$^{red}$.Zn$^{2+}$-σ$^{R}$ complex. RsrA$^{red}$.Zn$^{2+}$-σ$^{R}$ complex was incubated with a 2 mM mixture of BS$^{2}$G-d$_{0}$ and BS$^{2}$G-d$_{4}$ in 20 mM Hepes pH 7.5, 50 mM NaCl and 2 mM DTT at room temperature for 30 minutes. Cross-linking was stopped with 100 mM Tris pH 7.5. Samples were run on an SDS-PAGE and protein bands excised and analysed by LC-MS/MS. (Crosslinking with BS3-d$_{0}$/BS3-d$_{4}$ was done similarly). Crosslinks to all residues except RsrA K74 are coloured red. Crosslinks to RsrA K74 to σ$^{R}$ K71 is coloured yellow, RsrA K74 to σ$^{R}$ K86 is coloured black and RsrA K74 to σ$^{R}$ K149 is coloured blue. A single unique intramolecular cross-link within RsrA (56K-74K) was observed (XLR in the gel) which agrees with the solution structure of RsrA$^{red}$.Zn$^{2+}$. ***c***, Homology model of the RsrA$^{red}$.Zn$^{2+}$-σ$^{R}$ complex showing two possible conformations of helix IV. Model was built based on homology with ChrR-RpoE and RslA-σ$^{L}$ using Modeller V9.12. 100 models were generated and the lowest energy model shown. RsrA is coloured blue, σ$^{2}$ domain of σ$^{R}$ is coloured orange, σ$^{4}$ domain of σ$^{R}$ is coloured pink. Cylinders denote helices and are coloured as in *a*. Red lines denote the observed crosslinks. RsrA keeps the same fold in both the models except for the orientation of the final helix which could interact with either σ$^{2}$ or σ$^{4}$.

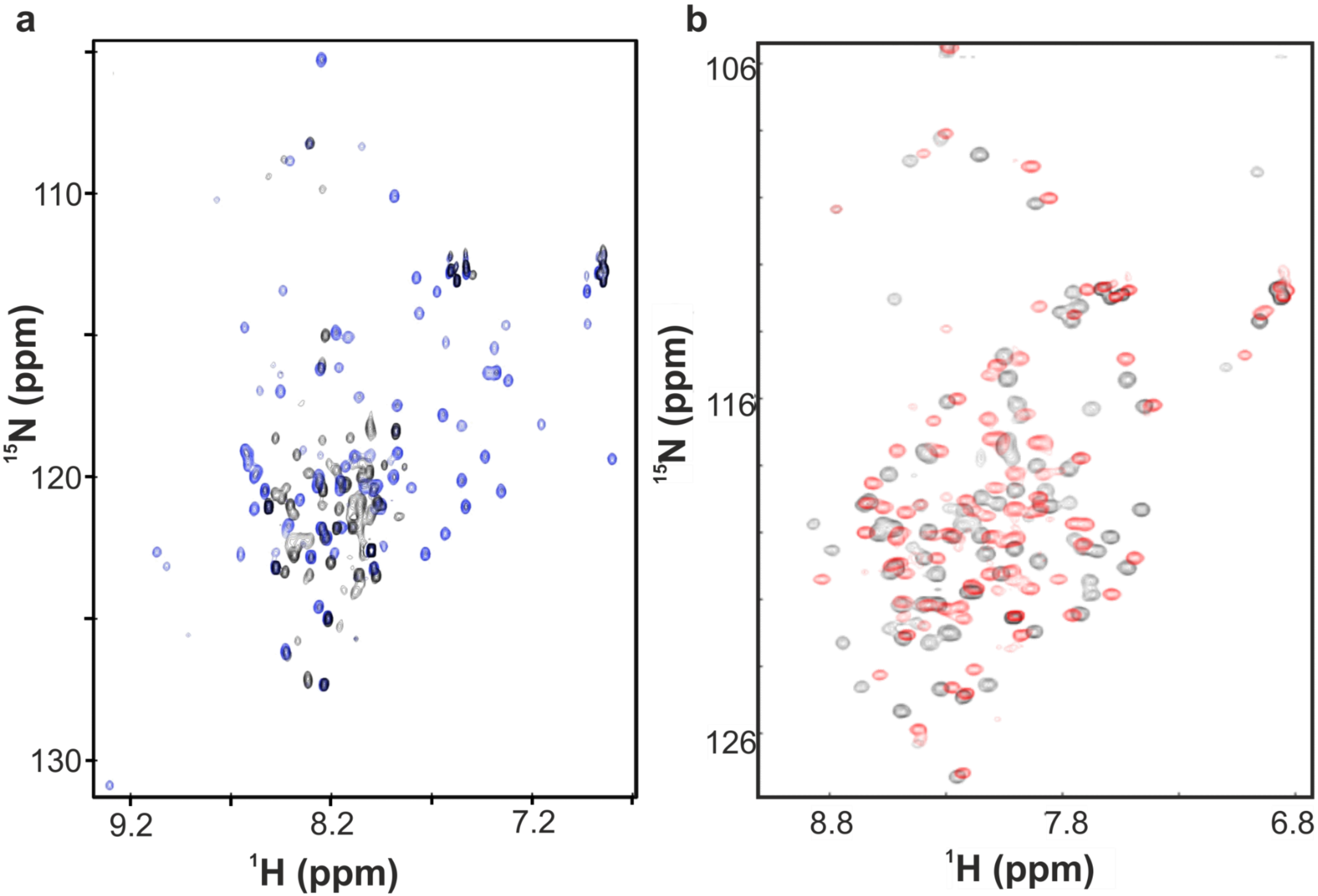


**Supplementary Figure 6. $^{1}H$-$^{15}N$ HSQC spectra of reduced and oxidized RsrA.** ***a***, $^{1}H$-$^{15}N$-HSQC spectrum of RsrA*$^{red}$ Cys41Ser in the absence of zinc (black) and bound to one equivalent of zinc (blue) in 20 mM Tris buffer pH 7.1 containing 5 mM DTT and 2 mM $ZnCl_2$. ***b***, Oxidised RsrA proteins containing either Cys11-Cys41 or Cys11-Cys44 disulfide bonds yield well-dispersed HSQC spectra characteristic of globular, folded proteins. Overlay of HSQC spectra of oxidized RsrA* Cys44Ser containing the Cys11-Cys41 disulfide bond (*black peaks*) and RsrA* Cys41Ser containing the Cys11-Cys44 disulfide bond (*red peaks*).

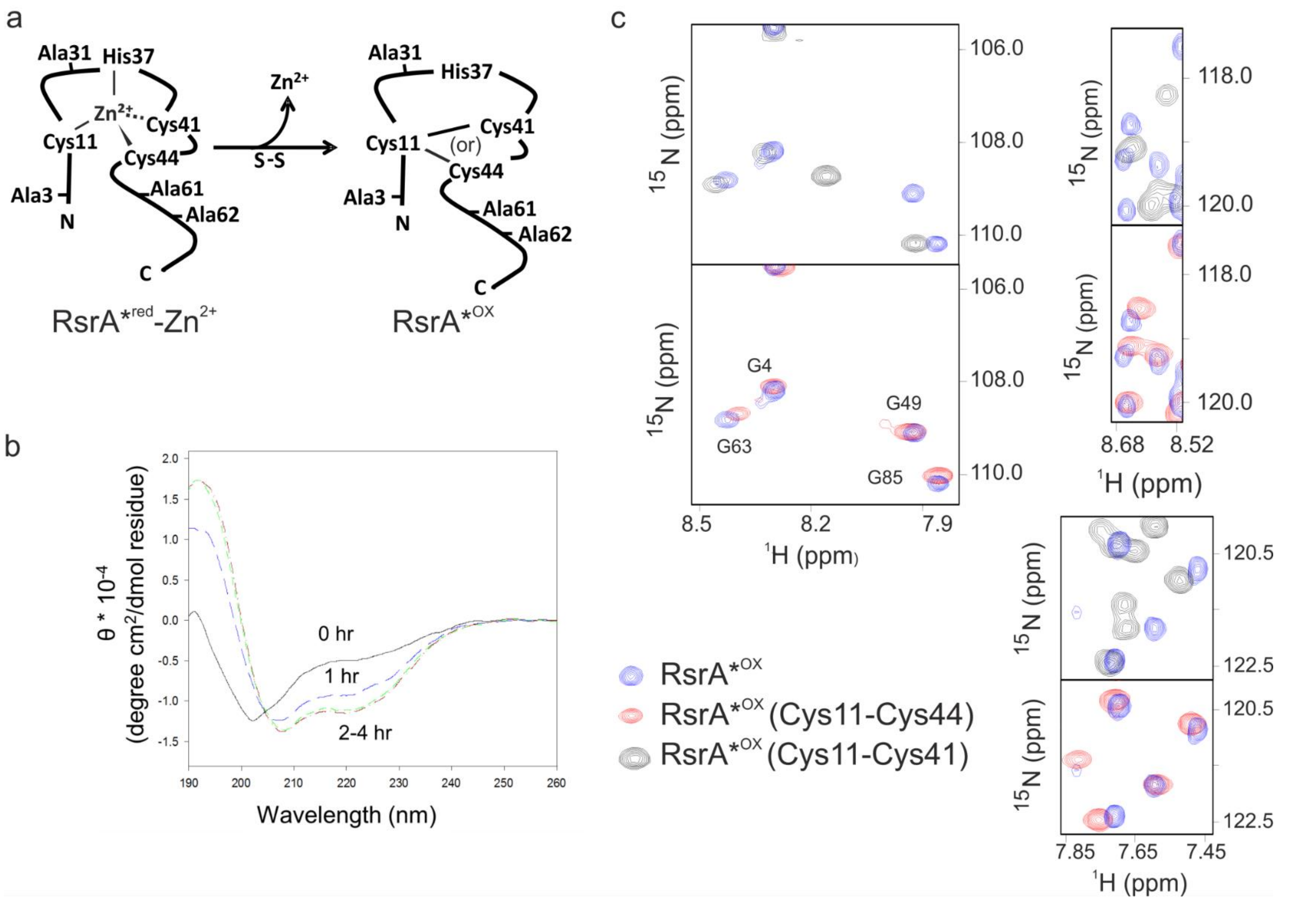


**Supplementary Figure 7. Identification of the most stable trigger disulfide bond in RsrA$^{ox}$.** ***a***, Schematic of RsrA showing the location of cysteine residues mutated to alanine in RsrA*, the identity of the zinc ligands and the trigger disulfide bond that forms in response to oxidation and which results in the loss of zinc. Previous studies have shown that the trigger disulfide is degenerate and forms between either Cys11 and Cys41 or Cys11 and Cys44. ***b***, Time-dependent changes in far UV-CD spectra (0.16 mg/ml) during formation of the RsrA* Cys11-Cys44 disulfide bond in RsrA* C41S as a result of air oxidation in 20 mM Tris pH 7.1, 25°C are similar to those previously reported for wild-type RsrA [25]. ***c***, Overlay of $^{1}H$-$^{15}N$-HSQC spectra of air-oxidized RsrA* (*blue peaks*), from which zinc had been removed, with $^{1}H$-$^{15}N$-HSQC spectra of RsrA*$^{ox}$ containing either the Cys11-Cys41 disulfide bond (*black peaks*) or the Cys11-Cys44 disulfide bond (*red peaks*). In each of the pure disulfided species of RsrA the third cysteine was mutated to serine to block mixed disulfide bond formation. The figure shows a comparison of three chemical shift regions demonstrating the overall similarity of the spectra of RsrA*$^{ox}$ with RsrA*$^{ox}$ containing the Cys11-Cys44 disulfide bond (lower panels in the three spectral regions shown) rather than the Cys11-Cys41 disulfide bond. Assignments for glycine residues in RsrA*$^{ox}$ Cys11-Cys44 are shown.

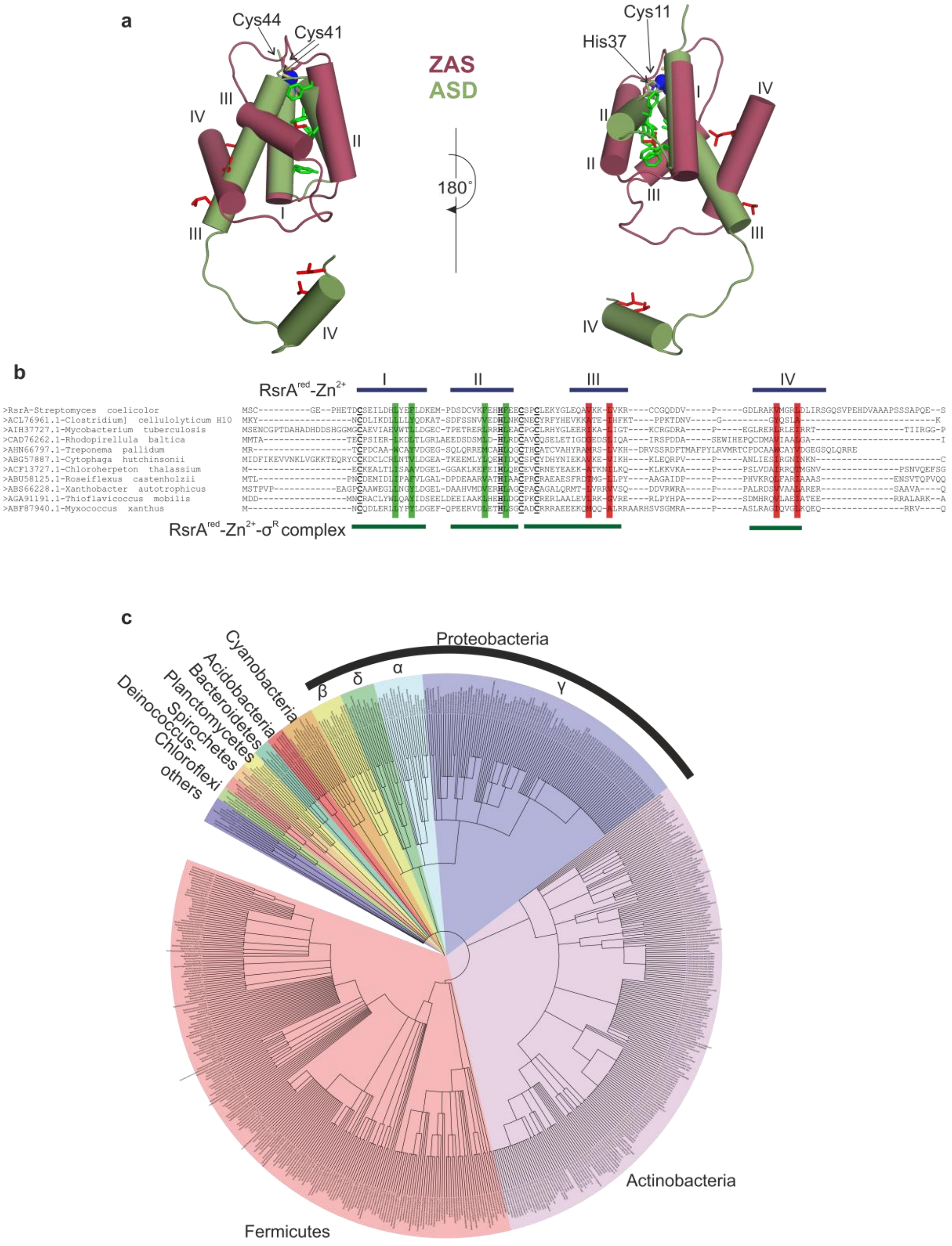


**Supplementary Figure 8. Comparison of the ZAS and ASD folds.** ***a***, Structural alignment of RsrA$^{red}$-Zn$^{2+}$(ZAS fold coloured *red*) and RsrA$^{red}$-Zn$^{2+}$ in complex with σ$^{R}$ (ASD fold coloured *green*). Helices are numbered from I to IV. Zinc is shown as a *blue sphere*, zinc ligands are shown as sticks and coloured based on atom type. Hydrophobic residues that form RsrA's core in both RsrA$^{red}$-Zn$^{2+}$ and the RsrA$^{red}$-Zn$^{2+}$-σ$^{R}$ complex are shown as *green sticks*. Hydrophobic residues that form RsrA's core in RsrA$^{red}$-Zn$^{2+}$ but also form interactions with the sigma factor in the RsrA$^{red}$-Zn$^{2+}$-σ$^{R}$ complex are shown as *red sticks*. The figure highlights how the ASD and ZAS folds represent different structural states of the same anti-sigma factor. ***b***, Structure-based sequence alignment of distantly related ZAS proteins from different phyla. *Streptomyces* and *Mycobacterium* belong to actinobacteria, *Clostridium* belongs to Firmicutes, *Rhodopirellula* under Planctomycetes, *Treponema* under Spirochaetes, *Cytophaga* under Bacteroidetes, *Chloroherpeto* under Chlorobi, *Roseiflexus* under Chloroflexi, *Zantobacter, thioflavicoccus and Myxococcus* under Proteobacteria. Helix limits of RsrA$^{red.}$Zn$^{2+}$ and RsrA$^{red}$Zn$^{2+}$-σ$^{R}$ complex are shown by *blue* and *green* horizontal bars, respectively. Hydrophobic residues are coloured as in *a*. Conserved hydrophobic residues in helices I and II (*green*) are involved primarily in stabilizing both the ZAS and ASD folds. In contrast, conserved hydrophobic residues in helices III and IV (*red*) have a dual role, stabilizing the ZAS fold in the absence of sigma factor and the protein-protein interaction with sigma factor. ***c***, Abundance of ZAS-ECF complexes in bacteria. The figure shows all organisms that have a ZAS domain close to an ECF. Searching for proteins similar to RsrA or ChrR with a zinc motif (C/H-x(23-26)-H-x(3)-C-x(2)-C) that lie less than 300 bases away from an ECF-σ homolog showed that more than 1100 genomes have ECF-ZAS pairs. Different sector colours denote different phyla.

| Protein \ Residues | 3 | 11 | 31 | 41 | 44 | 61 | 62 | $K_d$ (nM) | ΔH (kcal/mol) | ΔS (cal/mol/deg) |
|---|---|---|---|---|---|---|---|---|---|---|
| **RsrA (+) Wild type** | C | C | C | C | C | C | C | 0.78 ± 0.034 | -23.05 ± 1.10 | -38.70 ± 3.10 |
| **RsrA (-) Wild type** | C | C | C | C | C | C | C | 79.3 ± 4.2 | -16.26 ± 0.13 | -20.3 ± 4.34 |
| **RsrA* (+)** | A | C | A | C | C | A | A | 0.23 ± 0.031 | -19.64 ± 2.14 | -30.06 ± 4.70 |
| **RsrA* (-)** | A | C | A | C | C | A | A | 183 ± 7.1 | -11.51 ± 0.24 | -5.61± 0.71 |
| **RsrA* C41S (+)** | A | C | A | S | C | A | A | 557 ± 45.8 | -14.81 ± 0.33 | -19.50 ± 0.87 |
| **RsrA* C44S (+)** | A | C | A | C | S | A | A | ND | ND | ND |
| **RsrA* C11S (+)** | A | S | A | C | C | A | A | 79 ± 19 | -19.22 ± 0.91 | -29.87 ± 3.33 |
| **RsrA* C41S C44S (+)** | A | C | A | S | S | A | A | 297 ± 44.5 | -18.77 ± 1.23 | -31.10 ± 3.50 |
| **RsrA* C11S C44S (+)** | A | S | A | C | S | A | A | 185 ± 22.8 | -15.27 ± 0.25 | -18.60 ± 0.60 |
| **RsrA* C11S C41S (+)** | A | S | A | S | C | A | A | 240 ± 4.2 | -17.12 ± 0.54 | -25.45 ± 2.33 |
| **RsrA* C11H C41H C44H (+)** | A | H | A | H | H | A | A | 305 ± 2 | -15.76 ± 0.68 | -22.60 ± 0.70 |
| **RsrA V54A L57A (+)** | C | C | C | C | C | C | C | 105 ± 10.8 | -23.80 ± 1.48 | -46.2 ± 3.03 |
| **RsrA V75A L79A (+)** | C | C | C | C | C | C | C | 82.1 ± 2.24 | -15.5 ± 0.84 | -18.5 ± 2.16 |

Supplementary **Table 1: Affinity of wild type and mutant RsrA proteins for $\sigma^R$ in the presence (+) or absence (-) of $Zn^{2+}$ determined by ITC.** Table shows the identity of residues at each of the six cysteines in RsrA. Data for RsrA mutants of $\sigma^R$-contacting hydrophobic residues (final two rows) are also shown. RsrA* denotes RsrA in which all non-zinc ligating cysteines were mutated to alanine. See text for details. *ND*, not detected. Conditions for all experiments were 50 mM Tris pH 7.5 buffer containing 100 mM NaCl and 2 mM DTT, 35°C. No heats of binding could be detected at 25°C. Mean values are shown in the table along with standard deviations from three independent measurements.

| 25°C | $k_{on}$ ($M^{-1}s^{-1}$) | $k_{off}$ ($s^{-1}$) | $K_d$ (nM) from kinetics | $K_d$ (nM) from ITC |
|---|---|---|---|---|
| **+ $Zn^{2+}$** | $5.4 \pm 0.2 \times 10^6$ | $7.1 \times 10^{-4} \pm 2 \times 10^{-5}$ | 0.13 | ND |
| **- $Zn^{2+}$** | $11.49 \pm 1.69 \times 10^6$ | $0.286 \pm 0.051$ | 24.9 | ND |
| | | | | |
| **35°C** | | | | |
| **+ $Zn^{2+}$** | $8.9 \pm 1.4 \times 10^6$ | $0.0024 \pm 0.0004$ | 0.27 | $0.78 \pm 0.034$ |
| **- $Zn^{2+}$** | $17.3 \pm 1.3 \times 10^6$ | $1.08 \pm 0.01$ | 62.4 | $79.3 \pm 4.2$ |

**Supplementary Table 2: Association ($k_{on}$) and dissociation ($k_{off}$) rate constants for the RsrA-$\sigma^R$ complex in the presence or absence of $Zn^{2+}$ at 25°C and 35°C.** Mean values are shown along with standard deviations from three independent measurements. The kinetically-derived $K_d$s ($k_{off}/k_{on}$) at 35°C show reasonable agreement with those obtained by ITC, presented alongside for comparison. The affinity of the complex at 25°C in the presence of zinc is ~2-fold higher compared to 35°C. *ND*, not determined. Conditions for all experiments were 50 mM Tris pH 7.5 buffer containing 100 mM NaCl and 2 mM DTT.
